# Phase-matched multi-layer based polarisation-independent spot-size converter for silicon nanowire

**DOI:** 10.1038/s41598-019-48848-0

**Published:** 2019-08-26

**Authors:** Weifeng Jiang, B. M. Azizur Rahman

**Affiliations:** 1 College of Electronic and Optical Engineering, Nanjing University of Posts and Telecommunications, Nanjing, 210023 China; 2 Department of Electrical and Electronic Engineering, City, University of London, Northampton Square, London, EC1V OHB UK

**Keywords:** Integrated optics, Fibre optics and optical communications

## Abstract

The efficient coupling of optical power from a silicon nanowire (NW) to an optical fibre is challenging for both the quasi-TE and quasi-TM polarisations. Here, we propose a polarisation-independent spot-size converter (PI-SSC) based on phase-matched multi-layer waveguides for efficient coupling between a silicon NW and an optical fibre for both the polarisations. The fabrication process of the proposed PI-SSC is compatible with the complementary metal-oxide-semiconductor (CMOS) process. The optimisation for the proposed PI-SSC is studied by using a numerically efficient algorithm, combining a rigorous H-field based full-vectorial finite element method (VFEM) and the least squares boundary residual (LSBR) method. The simulation results show that using an eleven-layer based PI-SSC, the coupling losses between a silicon NW and a lensed fibre of radius 2 μm can be reduced to only 0.34 dB and 0.25 dB for the quasi-TE and quasi-TM polarisations, respectively. Furthermore, the output multi-layer is horizontally tapered, which further reduces the coupling loss for both the polarisations and the end face is easy to be polished.

## Introduction

Silicon photonic is an attractive platform for large-scale photonic integrated circuits (PICs), attributing to high refractive index contrast and complementary metal-oxide-semiconductor (CMOS)-compatible fabrication process^1,2^. Using highly matured CMOS process, silicon photonics fabricated on silicon-on-insulator (SOI) substrate can provide us with a low-cost and highly integrated photonic platform. Today, the wide use of silicon photonics is limited by the lack of highly efficient silicon lasers and high-performance modulators^3,4^. Besides these, the efficient coupling between a silicon nanowire (NW) with a small cross-section and a single-mode fibre (SMF) with a large area is also another major issue^5^. Although silicon photonic allows for large-scale integration of optical functions, it also severely complicates the interfacing with an SMF, as a result of the huge mismatch in the mode size between a silicon NW (≈0.2 μm^2^) and an SMF (≈80 μm^2^)^6,7^. Several approaches have been proposed to overcome this shortcoming, including a one-dimensional (1D) grating, an inverted taper, a 2D/3D taper, lens, a taper fibre, and a dielectric loaded plasmonic waveguide^8–15^. Although these approaches can improve the coupling efficiency, but often for only one of the polarisation states, either TE or TM mode of a silicon NW, can be coupled efficiently which may have limitation in many applications of optical communication systems. Although a silicon NW is always polarisation dependent due to the structural birefringence, however as an SMF cannot maintain its polarisation state, therefore, polarisation-diversity devices are often required. While researchers have paid considerable efforts to implement polarisation-diversity devices, but efficient polarisation-independent (PI) coupling is far from trivial.

In order to tackle the polarisation dependence in the coupling structures, various approaches such as the use of grating couplers (both 1D and 2D), inverted tapers (with or without polymer over-cladding), multi-stage tapers, and vertically bent Si NW (or elephant couplers) have been reported^7,16–22^. One approach could be the use of a grating coupler, either a 1D or 2D grating, which has been extensively studied and can be utilised in a PI-PIC with the efficient coupling for both the TE and TM polarisations^16–19^. Nevertheless, a 2D-grating coupler needs a relatively complicated optimisation to obtain high coupling efficiencies for both the polarisations. In addition, both 1D and 2D gratings inherently suffer from a trade-off between the coupling efficiency and bandwidth. Another approach could be the use of an inverted taper to transform a silicon NW mode to the mode of an upper polymer waveguide, matching the mode of a lensed fibre, which can yield a low-loss and polarisation insensitive coupler^20^. In order to obtain this high efficiency, a silicon taper with or without an upper polymer over-cladding needs to be tapered to an ultra-narrow tip with a width of nano-sized dimension. But, the facet of the inverted taper is needed to be polished for the coupling. Another approach could be the use of a double-stage taper, which can provide a PI and wavelength-insensitive mode conversion and can be fabricated by using the CMOS process^7^. However, the reported length of the taper is very long ~1000 μm and the coupling loss is at least −1.5 dB/facet for both the quasi-TE and quasi-TM polarisations, when coupling to a silicon rib waveguide with the size of 1.5 μm × 1.5 μm. A PI-3D taper has been reported to achieve a very low loss of <0.2 dB, but the fabrication process for this taper needs a complicated gray-scale lithography^21^. Recently, a PI “elephant coupler” has been proposed, in which the terminal of the silicon NW is bent vertically with a few-micron-scale curvature^22^. While this vertical coupler is promising for both the wafer-level testing and integration of optical components on chip surface, but the fabrication of a vertically curved silicon core is more challenging.

Recently, we have proposed a spot-size converter (SSC) incorporating phase-matched polycrystalline-silicon (Poly-Si) multi-layer, which can achieve a high coupling efficiency for the quasi-TE mode and can be fabricated by using the CMOS compatible process^23^. This SSC is based on the non-tapered structure, in which the phase-matching for the quasi-TE mode is achieved between the lower silicon NW and the upper Poly-Si multi-layer by adjusting the height and inner separation of the multi-layer section. Optical power in the silicon NW can be evanescently coupled to the phase-matched upper multi-layer after propagating along the SSC. The numerically simulated results show that the coupling losses can be reduced to 2.72 dB and 0.34 dB when coupled to an SMF and a lensed fibre of radius 2 μm, respectively for the quasi-TE mode^23^.

Here, we extend our original concept of the multi-layer based SSC to a PI operation, which can provide the efficient coupling between a silicon NW and an optical fibre for both the quasi-TE and quasi-TM polarisations. The orthogonally polarised lights from a silicon NW can be coupled to two upper multi-layers separately and then combined together at the end of the PI-SSC. In this case, vector modes and supermodes of the isolated and composite waveguides are calculated by using the rigorous **H**-field based full-vectorial finite element method (VFEM)^24,25^. Confinement factors of the supermodes are studied and phase-matching conditions are determined for both the quasi-TE and quasi-TM modes. Subsequently, the supermode coefficients and power transfer efficiency for both the polarisations are studied by using the least squares boundary residual (LSBR) method^26,27^. The coupling losses for both the polarisations between a multi-layer based SSC and an SMF are calculated by using the VFEM and LSBR approaches based on our in-house codes.

## Results

### Schematic and principle

**Figure 1:**
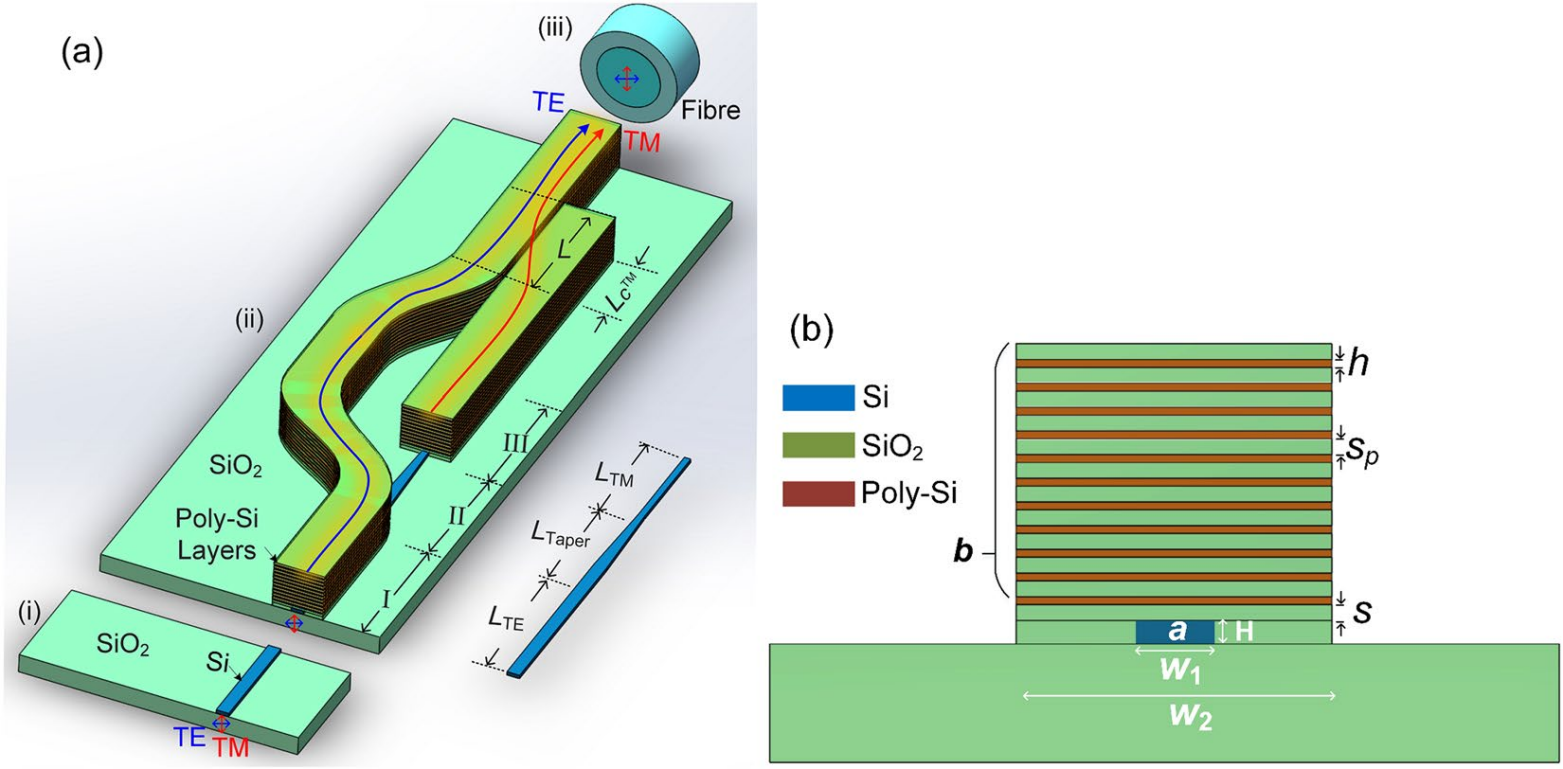
Schematic of polarisation-independent SSC based on the multi-layer. (**a**) Schematic diagram for coupling process. (**b**) Cross-section of the multi-layer structure.

The schematic diagram of the PI-SSC based on the multi-layer is shown in Fig. 1(a), consists of two multi-layer sections on an SOI platform and an untapered silicon NW followed by a taper and then by another untapered silicon NW (untapered-tapered-untapered silicon NW). The taper length and coupling lengths of the quasi-TE and quasi-TM polarisations are taken as *L*_taper_, *L*_TE_, and *L*_TM_, respectively. The cross-section of the PI-SSC is shown in Fig. 1(b), consists of a lower silicon NW, *a* and an upper multi-layer waveguide, *b*. For the proposed PI-SSC, the primary waveguide, *a*, is phase matched with the second waveguide, *b*, to evanescently couple optical power from the lower silicon NW to the upper multi-layer waveguide. The proposed PI-SSC can be fabricated by using the following steps: (i) the lower silicon NW, *a*, is fabricated based on the plasma enhanced chemical vapour deposition (PECVD) and reactive ion etching (RIE). After step (i) for the fabrication of the silicon NW, the silicon NW is subsequently cladded with a PECVD SiO_2_. As the conformal process would create a bump above the silicon NW, so the wafer is needed to be planarised to the NW level by using a chemical mechanical polishing (CMP) tool and a KOH and silica solution, as described in ref.^28^. Subsequently, multiple layers of Poly-Si and SiO_2_ needed for both the quasi-TE and quasi-TM polarisations can be deposited simultaneously by utilising the PECVD. The whole stack can be etched together, in which only one extra mask is needed for the fabrication of the multi-layer sections.

It can be observed from Fig. 1(b) that the height and width of the multi-Poly-Si layer are taken as *h* and W_2_, respectively. In this study, the width of the upper array, W_2_, is taken as 6 μm to couple with a standard SMF and a lensed fibre, respectively. The radius, core, and cladding refractive indices of a standard SMF are taken as 4.225 μm, 1.4552, and 1.45, respectively. With a same normalised frequency, V, of the SMF, the radius, core, and cladding refractive indices of a lensed fibre are taken as 2 μm, 1.4731, and 1.45, respectively. The separation between the upper array and the lower silicon NW with the size of W_1_ × H is denoted by *S*, which has the impact on the crosstalk and coupling length. The inner separation of the multi-layer is denoted by *S*_p_, which can be optimised to achieve the maximum effective area (*A*_*eff*_) for the multi-layer sections. The size of the silicon NW is taken as W_1_ × H = 400 nm × 220 nm, considered as a typical silicon NW. The refractive indices of the Poly-Si, silicon, and silica are taken as 3.48, 3.47548, and 1.46, respectively at the operating wavelength of 1.55 μm. In this case, an eleven-layer based PI-SSC is optimised to achieve the maximum coupling efficiency between a silicon NW and an optical fibre by using the rigorous **H**-field based VFEM and the LSBR method.

**Figure 2:**
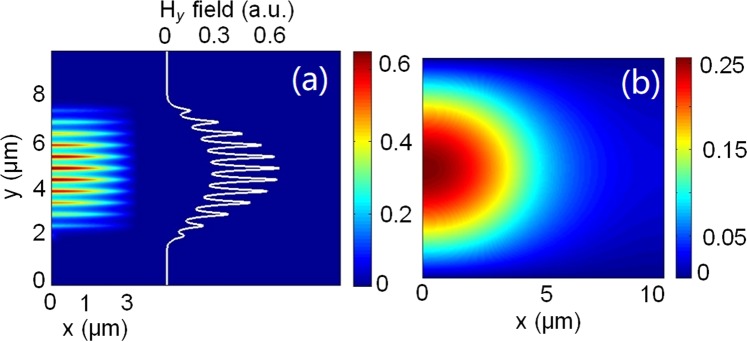
(**a**) The quasi-TE modal field profile at *z* = *L*_TE_ for the proposed PI-SSC and (**b**) the quasi-TE modal field profile of an SMF.

In order to achieve an eleven-layer based PI-SSC, the phase-matching condition for the quasi-TE mode has recently been reported in ref.^23^, in which the height, *h*, and inner separation, *S*_p_, of the eleven-layer were determined to be 105 nm and 400 nm, respectively. Although a higher coupling efficiency can be obtained with a larger number of the multi-layer, the fabrication would be more demanding. Hence, an 11-layer is considered in this case, for which the coupling efficiency is reasonably high, yet the proposed structure is relatively easy to fabricate. The separation between the upper 11-layer array and the lower silicon NW was taken as 200 nm. The maximum transfer of optical power from the lower silicon NW to the upper multi-layer was 95.2% at *z* = *L*_TE_ = 158.67 μm for the quasi-TE mode. The modal field profile of the quasi-TE mode at *z* = *L*_TE_ = 158.67 μm is shown in Fig. 2(a) and that of the SMF is shown in Fig. 2(b). Only half of the modal field is shown for each structure due to the structural symmetry of both PI-SSC and SMF. It can also be noted from Fig. 2(a) that the modal field profile of the quasi-TE mode was significantly expanded by utilising the 11-layer based SSC. The total coupling losses for the quasi-TE mode are only 2.72 dB and 0.34 dB, when coupled to a standard SMF and a lensed fibre of radius 2 μm, respectively. The total coupling loss is the sum of the losses, including the first butt-coupling of optical power from the input NW to the lower silicon NW of the SSC at *z* = 0 followed by the evanescent coupling from the lower silicon NW to the upper 11 Poly-Si layers at exactly *z* = *L*_*c*_, and finally second butt-coupling loss between the output of the SSC and optical fibre. The reflected powers at the end of the SSC-SMF and SSC-lensed fibre were 6.8% and 1.8%, respectively. Therefore, in Section I, the height and inner separation of the upper multi-layer can be adjusted to achieve the phase matching with the lower silicon NW for the quasi-TE polarisation. However, as the phase matching is not satisfied for the quasi-TM polarisation due to effective indices of the silicon NW and the upper 11-layer were of significantly different values, 1.7107 and 1.6355, respectively, the quasi-TM mode continues its propagation along the silicon NW.

### Characterization of mode properties

**Figure 3:**
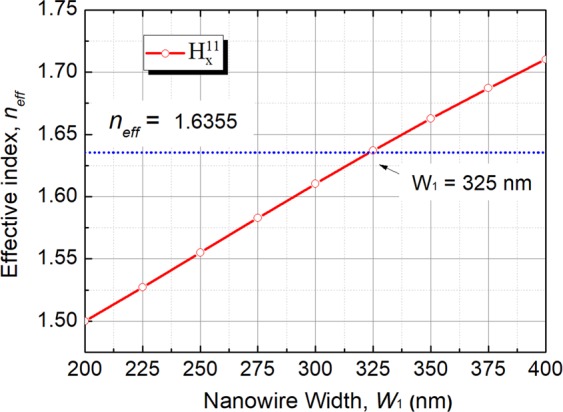
Variation of the effective index, *n*_*eff*_ with the width of the silicon NW, W_1_ for the quasi-TM polarisation.

In order to achieve the phase matching for the quasi-TM mode, a novel approach using an untapered-tapered-untapered silicon NW is introduced as a lateral mode-size converter for the quasi-TM mode, shown in Fig. 1(a). After achieving the phase-matching condition for the quasi-TE mode, the height, *h*, inner separation, *S*_p_, coupling separation, *S*, and height of the silicon NW, H, were kept fixed during the design of the coupling structure for the quasi-TM mode in Section III, which can thereby simplify the requirement of the planar lightwave circuit (PLC) fabrication. In order to study the phase matching for the quasi-TM mode in Section III, the effective index of the isolated silicon NW for different width is calculated by using the VFEM to identify the necessary W_1_ for the phase matching. Variation of the effective index, *n*_*eff*_ with the width of the silicon NW, W_1_ for the quasi-TM mode is shown in Fig. 3 by a solid red line. It can be noted that the effective index of the fundamental quasi-TM, ${{\rm{H}}}_{x}^{{\rm{11}}}$ mode of the isolated 11-layer is equal to *n*_*eff*_ = 1.6355 and shown by a horizontal dashed blue line. It can also be noted that the phase matching to the isolated array can be attained when NW width, W_1_ = 325 nm. Although the phase matching is shown for the isolated waveguides, it is essential to be studied for the coupled guides, in which the width for the phase matching may be shifted considerably.

**Figure 4:**
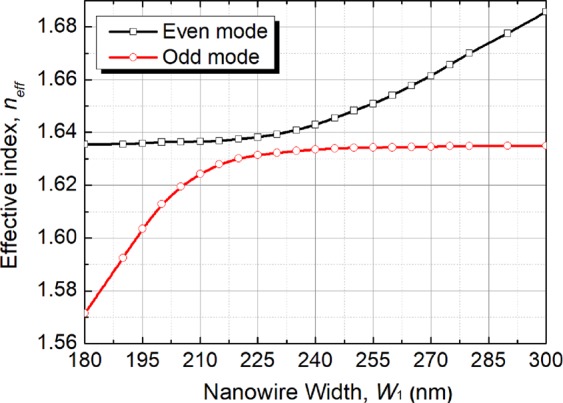
Variations of the effective index, *n*_*eff*_ for even and odd modes with the width of the silicon NW, W_1_ for quasi-TM polarisation.

Next, the supermodes are calculated by using the **H**-field based VFEM for the coupled structure. Variations of the effective index, *n*_*eff*_ of the even-like and odd-like quasi-TM supermodes with the width of the silicon NW, W_1_ are shown in Fig. 4 by solid black and red lines, respectively. The effective index of the ${{\rm{H}}}_{x}^{{\rm{11}}}$ mode of the upper multi-layer (almost constant) is denoted by the horizontal line sections of these two curves. It can be noted that the effective indices of the ${{\rm{H}}}_{x}^{{\rm{11}}}$ mode of the lower silicon NW is represented by the slanted line sections of these two curves, which are increased with the width, W_1_. It can be observed from Fig. 4 that when the width of the silicon NW, W_1_ ~ 225 nm, these two effective indices become closer and the phase difference between these modes will be smallest. In this situation, two isolated modes become degenerate, get mixed up and form two supermodes. But, these two curves do not cross each other and both supermodes undergo a transformation around this anti-crossing region.

**Figure 5:**
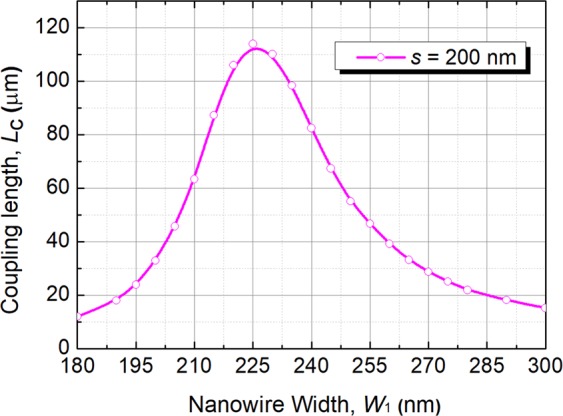
Variation of the coupling length, *L*_c_ with the width of the silicon NW, W_1_ for quasi-TM polarisation.

Next, the coupling length can be calculated using the formula as *L*_c_ = π/(*β*_even_ − *β*_odd_) where *β*_even_ and *β*_odd_ are the propagation constants of the even-like and odd-like supermodes, respectively^23^. Variation of the coupling length with the width of the silicon NW, W_1_ is shown in Fig. 5 for *S* = 200 nm. It can be noted that when the phase matching was achieved with the width of the silicon NW, W_1_ = 225 nm, the coupling length shows a peak value of *L*_TM_ = 113.9 μm. It can also be noted that the phase-matching condition for two non-identical guides shifts significantly from that for two isolated guides due to the unequal loading of each other.

**Figure 6:**
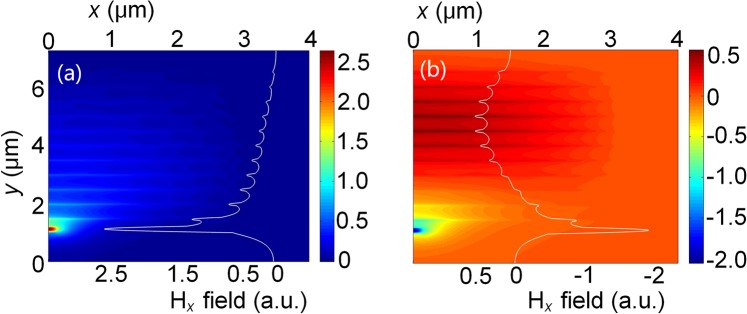
Hx field profiles of the phase matched ${{\rm{H}}}_{x}^{{\rm{11}}}$ **(a)** even-like and **(b)** odd-like supermodes.

**Figure 7:**
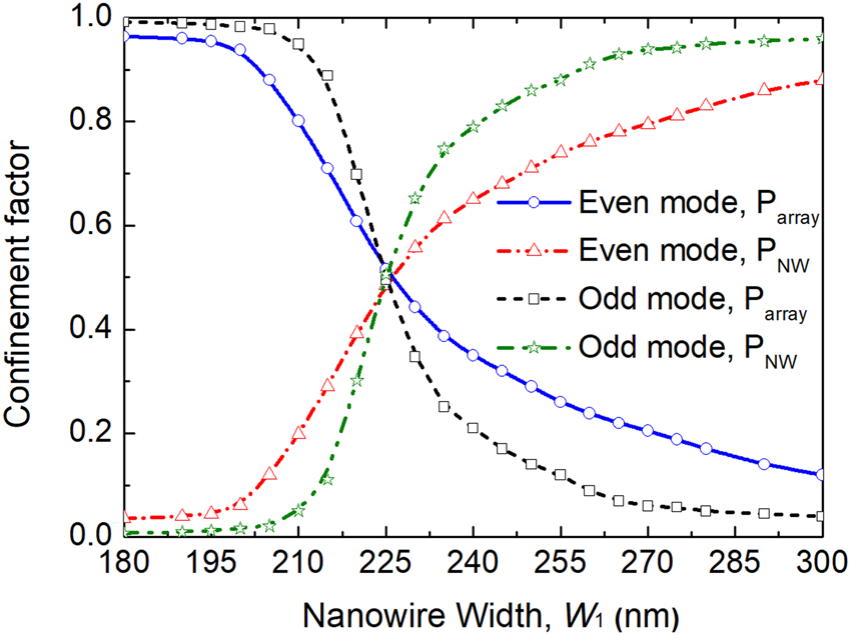
Variations of the confinement factor with the width of the silicon NW, W_1_ for the quasi-TM polarisation.

**Figure 8:**
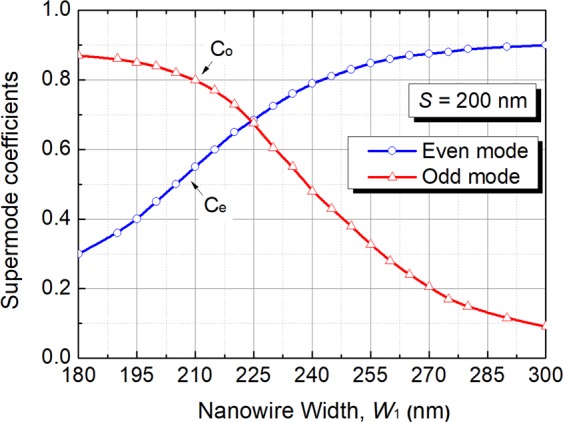
Variations of the of even and odd supermode coefficients, C_e_ and C_o_, respectively, with the width of the silicon NW, W_1_ for the quasi-TM polarisation.

H_x_ field profiles of the phase-matched ${{\rm{H}}}_{{\rm{x}}}^{{\rm{11}}}$ even-like and odd-like supermodes are shown in Fig. 6. It can be noted that these supermodes are not strictly even- or odd-type since two guides were non-identical. Although maximum amplitudes of the upper array and the lower silicon NW are different as their core sizes were also different, but nearly equal powers are distributed in these two non-identical guides, which can be verified by the confinement factors and excited supermode coefficients, shown in Figs 7 and 8, respectively. Similarly, for the odd-like supermode shown in Fig. 6(b), nearly equal powers are also distributed in two non-identical guides. However, the magnitudes of the upper array and the lower silicon NW have opposite signs and the amplitude of each layer decreases from the centre of the array to the edge of the array, shown with tapering amplitudes.

The confinement factor in any particular area normalised to the total power, which can be calculated by integrating the Poynting vector over a given region as^29^:

1 $${{\rm{\Gamma }}}_{i}=\frac{{\rm{\iint }}{}_{{{\rm{\Omega }}}_{i}}{\{{{\bf{E}}}^{\ast }\times {\bf{H}}\}}_{z}dxdy}{{\rm{\iint }}{}_{{{\rm{\Omega }}}_{\infty }}{\{{{\bf{E}}}^{\ast }\times {\bf{H}}\}}_{z}dxdy}$$

where **E** and **H** are the electric and magnetic fields of the calculated mode, Ω_i_ is the area of any particular guide and Ω_∞_ is the total area. The confinement factors of each guide are calculated by using the VFEM. Variations of the confinement factor with the width of the silicon NW, W_1_ are shown in Fig. 7. It can be noted that the confinement factors of the upper array and the lower NW for both even-like and odd-like supermodes are equal to 1/2, when the phase matching is achieved. This confirms that optical powers in two guides are equal for the phase-matched supermodes. As the width of the silicon NW, W_1_ is decreased, most of the even-like supermode power moves into the upper array and for the odd-like supermode, most of the power remains in the silicon NW. When the silicon NW, W_1_ gets larger, the even-like supermode power gets more confined in the silicon NW and for the odd-like supermode, most of the power is in the upper array. For weakly coupled identical waveguides, coefficients of two excited supermodes can be equal to $1/\sqrt{2}$, when an incident wave is launched into anyone of these two waveguides. However, for strongly coupled and/or non-identical waveguides, coefficients of two supermodes may not be equal to 0.707, which can cause incomplete transfer of optical power with an increased crosstalk. Especially, supermode coefficients would be highly unequal for non-identical waveguides when they are not phase-matched. To study the phase matching for two non-identical coupled guides, supermode coefficients for even-like and odd-like supermodes are calculated by using the LSBR method. The LSBR method was used here to calculate the power transfer efficiency by imposing the continuity of the transverse fields at the junction interface in a least-squares sense and obtain the supermode coefficients by considering both the guided and radiated modes at the discontinuity interface. For a discontinuity interface, both guided and radiated modes are generated to satisfy the necessary boundary conditions. In case of the butt coupling between a single-mode guide and a directional coupler section, both the supermodes are excited at the junction. For weakly coupled and phase matched guided, their power are equal. But, this is not true, particularly for nonidentical or strongly coupled waveguides, thereby the LSBR finds these excited coefficients of the supermodes^27^. Variations of even-like and odd-like supermode coefficients (C_e_ and C_o_) with the width of the silicon NW, W_1_ are shown in Fig. 8 by the solid blue and red lines, respectively. It can be noted that as the width of the silicon NW, W_1_ is increased, the coefficient of the even-like supermode (C_e_) is monotonically increased, whereas the coefficient of the odd-like supermode (C_o_) is monotonically reduced. The even-like and odd-like supermode coefficients have very similar values of 0.69 and 0.68, respectively, under the phase-matching condition with W_1_ = 225 nm. In this case, these values are also nearly equal to $1/\sqrt{2}$, and so two supermodes carry approximately half of the total power. When the width of the silicon NW, W_1_ is far away from the phase matching, two supermode coefficients are considerably different.

### Operation and bandwidth

It can be noted from the schematic diagram shown in Fig. 1 that the coupling process consists of three sections (i), (ii) and (iii). At *z* = 0, the section (i) is butt-coupled with the section (ii), where an input silicon NW is butt-coupled to the lower silicon NW of the PI-SSC. As the quasi-TE mode is phase matched in Section I, optical power of the quasi-TE mode would be evanescently coupled from the lower silicon NW to the upper multi-layer at the end of the Section I and then propagates along the upper multi-layer guide. Meanwhile, the quasi-TM mode continues to propagate along the Section I through the NW without coupling to the layered guide due to the lack of the phase matching and this mode can be transformed along Section II by the tapered waveguide. Subsequently, the phase matching between the lower silicon NW and the upper multi-layer guide can be achieved for the quasi-TM mode in Section III. Thus, the quasi-TM mode would also be evanescently coupled from the lower silicon NW to the upper multi-layer guide at the end of the Section III.

**Figure 9:**
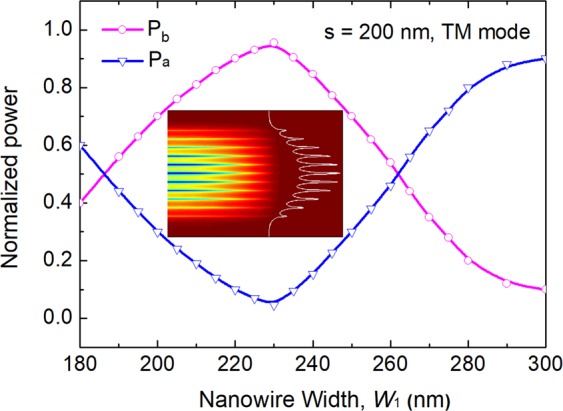
Variations of the normalised power transfer efficiency with the width of the silicon NW, W_1_ for the quasi-TM polarisation. The inset is the modal field at the quasi-TM output port.

Next, the power transfer efficiency is studied by using the LSBR method for the quasi-TM mode in Section III. Variations of the normalised power transfer efficiency for the quasi-TM mode are calculated with the width of the lower silicon NW, W_1_, shown in Fig. 9. Here, P_a_ and P_b_ are optical powers remaining in the lower silicon NW and coupled to the upper multi-layer guide, respectively, which are shown by the solid blue and red lines, respectively. It can be noted from Fig. 9 that at *z* = *L*_TM_ = 113.9 μm, the power transfer efficiency of the quasi-TM mode is 95.5% for the phased-matched NW width of 225 nm. The output modal field profile of the quasi-TM mode is shown as an inset in Fig. 9. It can be observed that the field profile of the quasi-TM mode is expanded by utilising the multi-layer based PI-SSC, in which the *A*_*eff*_ of the quasi-TM mode is converted from 0.58 μm^2^ of the lower silicon NW to 63.8 μm^2^ of the upper multi-layer. It can be noted from Fig. 9 that as W_1_ reduces from 243 nm to 208 nm, the resulting deterioration of the normalised power transfer efficiency was less than 20%. However, the width, W_1_ of the tapered section reduced from 400 nm to 225 nm, as a narrower width of the lower silicon NW in Section III will cause additional loss. Hence, the lower width, W_1_ should be kept in between 225 nm to 243 nm to keep the output power loss less than 1 dB for the quasi-TM mode. Next, the coupling efficiency between the quasi-TM mode in the output of the PI-SSC and an SMF/lensed fibre is calculated by using the LSBR method. For the coupling to a standard SMF, the coupling efficiency of 59.6% can be achieved for an 11-layer based PI-SSC. However, the coupling efficiency for the quasi-TM mode could be improved to 98.8% when coupled to a lensed fibre with a radius of 2 μm. The total coupling losses are 2.45 dB and 0.25 dB for the coupling to an SMF and a lensed fibre, respectively. The reflected powers from the PI-SSC and fibre junction are 5.4% and 0.9% when coupled to these two kinds of fibres, respectively. Although the phase matching for the quasi-TM mode cannot be achieved in Section I, there may be a small amount of power coupled to the upper array due to the incomplete mismatch and this power transfer has been calculated as only 0.63% at the end of the Section I (*z* = *L*_TE_).

**Figure 10:**
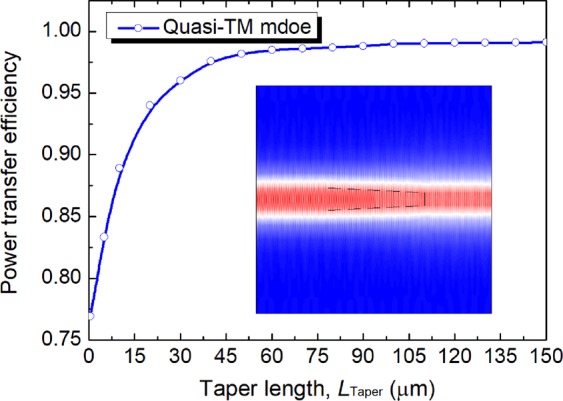
Variation of the power transfer efficiency with the taper length, *L*_taper_. The inset is the propagation modal field for *L*_taper_ = 60 μm.

As shown in Fig. 1, silicon NW can be adiabatically tapered to reduce the waveguide width in Section II. The widths of the input and output cross-sections for the taper are 400 nm and 225 nm, as discussed earlier, necessary for the phase matching of the quasi-TM mode. Variation of the power transfer efficiency with the taper length, *L*_taper_ is calculated by using the commercial COMSOL Multiphysics and shown in Fig. 10. It can be noted that the power transfer efficiency is higher than 98.5% when the length was greater than 60 μm. The propagating modal field for the quasi-TM mode is shown as an inset in Fig. 10, which indicates that the field profile has been adiabatically transformed by using the linear taper.

**Figure 11:**
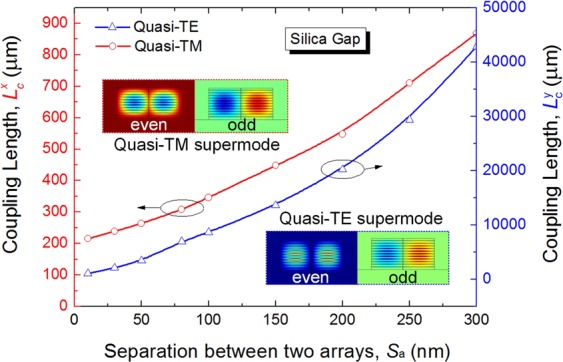
Variations of the coupling length with the separation between two arrays for the quasi-TE and quasi-TM modes, respectively.

In order to combine the quasi-TE and quasi-TM modes of two multi-layer sections, a polarisation combiner based on a directional coupler is introduced as shown in Fig. 1(a). Variations of the coupling length with the separation between two arrays are shown in Fig. 11 for both the quasi-TE and quasi-TM modes. The coupling lengths, ${L}_{{\rm{c}}}^{y}$ of the quasi-TE mode and ${L}_{{\rm{c}}}^{x}$ of the quasi-TM mode are shown by the red and blue lines, respectively (note: different scales are used). When the gap between two arrays is taken as the silica, it can be noted that the coupling length of the quasi-TE mode is nearly 30 times larger than that of the quasi-TM mode. For a typical silicon waveguide, since its width is much bigger than the height, so the effective index of the TE mode is higher than that of the TM mode. As a result TE modes are more confined, and resulting the coupling length of the TE mode is significantly larger. This large difference enables the power of the quasi-TM mode to be completely coupled to the array of the quasi-TE mode, whereas the power coupling of the quasi-TE mode to the array of the quasi-TM mode can be neglected. We choose the separation, *S*_a_ = 100 nm as an example to estimate the characteristic of the combiner. In addition, any wider separation can also be chosen but this would increase the coupling length. When *S*_a_ = 100 nm, the coupling lengths of the quasi-TE and quasi-TM modes are ${L}_{{\rm{c}}}^{y}$ = 8679 μm and ${L}_{{\rm{c}}}^{x}$ = 346 μm, respectively. The even and odd supermodes of the quasi-TE and quasi-TM modes are shown as two insets in Fig. 11. When the length of the polarisation combiner, *L* is taken as *L* = ${L}_{{\rm{c}}}^{{\rm{TM}}}$ = ${L}_{{\rm{c}}}^{x}$ = 346 μm, the power of the quasi-TM mode can be completely coupled to the array of the quasi-TE mode, shown as the left array in Fig. 1(a). In order to calculate the power of the quasi-TE mode coupled to the array of the quasi-TM mode, the power transfer efficiency can be calculated as being only 0.39%, based on the formula as *η* = sin^2^(${L}_{{\rm{c}}}^{x}$*π/2 ${L}_{c}^{y}$).

The coupling characteristics of the multi-layer based PI-SSC are summarised in Table 1 for both the quasi-TE and quasi-TM modes. The Junction 1 is the butt-coupling location from the lower silicon NW to the start of PI-SSC section and the Junction 2 is the butt-coupling location between the output of the PI-SSC and an SMF or a lensed fibre. It can be noted from Table 1 that the total coupling losses are 2.72 dB and 2.45 dB for the quasi-TE and quasi-TM polarisations, respectively when coupled to an SMF. But, for coupling to a lensed fibre of radius 2 μm, the total coupling losses are substantially reduced to 0.34 dB and 0.25 dB for the quasi-TE and quasi-TM polarisations, respectively. As a result of the reduction of the mode-size mismatch by using this multi-layer based PI-SSC, the reflectance can also be reduced to 1.8% and 0.9% for the quasi-TE and quasi-TM polarisations, respectively. In practice, the reflectance can be further reduced by using the index-matching oil between the PI-SSC and a lensed fibre. Considering the loss of the polarisation combiner for the quasi-TE mode (0.39%), the total loss of the quasi-TE mode for the PI-SSC is only 0.36 dB. Similarly, the total loss of the quasi-TM mode for the PI-SSC is 0.35 dB, considering the coupled power to the upper array in Section I (0.63%) and the power transfer loss in the untapered-tapered-untapered silicon NW (1.5%). In the numerical simulations, the material loss of the polysilicon was not included. However, for the polysilicon waveguides, fabricated via high temperature (1100 °C) anneals and special hydrogen plasma passivation steps, show a low loss of 9 dB/cm^30^. Therefore, the additional loss can be estimated to be ~0.6 dB for the proposed PI-SSC when the material loss of the polysilicon waveguide is included.

**Table 1:** Coupling characteristics of the multi-layer based PI-SSC.

Polarisation	NW (W_1_ × H)	Multi-layer			Coupling Length (μm)	Coupling efficiency		Total Coupling Loss (dB)	Reflectance
		*h* (nm)	*S*_p_ (nm)	*S* (nm)		Junction 1	Junction 2		
Quasi-TE	400 nm × 220 nm	105	400	200	*L*_TE_ = 158.67	95.2%	56.1%	2.72	6.8%
							*97.1%	*0.34	*1.8%
Quasi-TM	225 nm × 220 nm				*L*_TM_ = 113.9	95.5%	59.6%	2.45	5.4%
							*98.8%	*0.25	*0.9%

^*^Coupling between an eleven-layer based PI-SSC and a lensed fibre of radius 2 μm.

**Figure 12:**
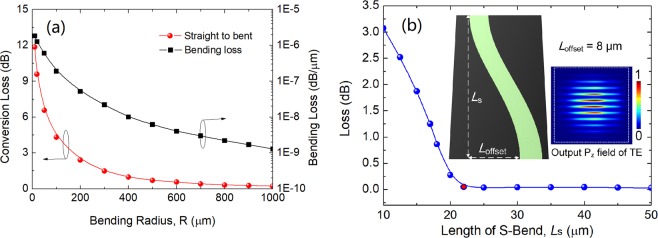
(**a**) Variations of the mode conversion loss (left y-axis) and bending loss (right y-axis) with the bending radius for the quasi-TE polarisation of the layered waveguide. **(b)** Variation of the loss with the length of the S-bend layered waveguide. The insets show the structure of the S-bend layered waveguide and the output P_z_ field of the quasi-TE polarisation.

The total length of the proposed PI-SSC is calculated based on the formula: *L*_total_ = *L*_TE_ + *L*_Taper_ + *L*_TM_ + *L* = 678.57 μm, where *L*_TE_, *L*_Taper_, *L*_TM_, and *L* are the lengths of the Sections I, II, III, and the polarisation combiner, respectively. It can be noted from Fig. 1(a) that a section of two successive S-bend layered-waveguides is implemented for the quasi-TE polarisation, while that is not required for the quasi-TM polarisation. Hence, the bending loss and conversion loss of the bending layered-waveguide are calculated for the quasi-TE polarisation. Variations of the mode conversion loss (left y-axis) and bending loss (right y-axis) with the bending radius are shown in Fig. 12(a). It can be noted that the bending loss is ultra-small as the lateral index contrast is high, so a compact bend can be used. However, the conversion loss between the straight and bent layered-waveguides is higher than the bending loss due to the mode-field mismatching. It can be noted from Fig. 12(a) that a radius of 800 μm is required for the mode conversion loss of 0.3 dB. In addition, a lateral offset between the bent and straight waveguides at these junctions can be introduced to reduce the mode conversion loss further. Variation of the mode conversion loss with the offset between the bent and straight layered-guides is calculated. An optimised offset of 0.48 μm can achieve a low mode-conversion loss of only 0.07 dB for the bent radius of 800 μm. Nevertheless, as the S-bend layered-waveguide is considered in this case, the necessary length can be shortened. The loss of the S-bend layered-waveguide is calculated with its length by using the 3D full-vectorial finite difference time domain (3D-FV-FDTD) method, as shown in Fig. 12(b). The structure of the proposed S-bend layered-waveguide is shown as an inset, in which the length and offset are denoted by *L*_S_ and *L*_offset_, respectively. In this case, the offset is chosen as *L*_offset_ = 8 μm, which can enable a 2 μm separation between two adjacent layered-waveguides, thereby avoid the unnecessary coupling. It can be noted from Fig. 12(b) that a compact length of *L*_S_ = 22 μm can be achieved for the loss < 0.05 dB and the related output Poynting vector, P_z_ field of the quasi-TE mode is shown as an inset. As two successive S-bend layered-waveguides are implemented, the total length of the S-bend section is 44 μm. It should be noted that the taper length of *L*_taper_ = 60 μm is longer than the total length of the S-bend section. Hence, the length of the Section II should be determined by the taper length and the overall length can be 678.57 μm. In this case, an ideal directional coupler was considered for the calculation of the coupling length based on the LSBR method. As the optimised S-bend layered-waveguide was calculated to be *L*_S_ = 22 μm, the overlapped length between the lower silicon NW and upper layered-waveguide should be less than 5 μm, which is reasonably small compared with the coupling length of *L*_TE_ = 158.67 μm. The 3D-FV-FDTD method can also be used to obtain the accurate results for the combination of both the straight and bending waveguides, but this would be too expensive computationally.

**Figure 13:**
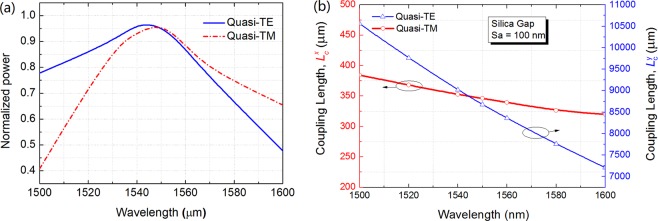
Variations of the **(a)** normalised power and **(b)** coupling length with the operation wavelength.

The wavelength dependence of the proposed PI-SSC is calculated by using the LSBR method. Variations of the normalised power with the wavelength are shown in Fig. 13(a) for both the quasi-TE and quasi-TM modes, in which the power transfer efficiency is the transfer of optical power from the lower silicon NW to the upper multi-layer at *z* = *L*_TE_ or *L*_TM_, respectively. It can be noted that the coupling efficiency as high as 95.2% and 95.5% for the quasi-TE and quasi-TM modes, respectively can be obtained at 1550 nm wavelength. The 1 dB bandwidth is 63 nm from 1504 nm to 1567 nm for the quasi-TE mode and this is 46 nm from 1526 nm to 1572 nm for the quasi-TM mode. Therefore, the combined 1 dB bandwidth is 41.4 nm from 1526 nm to 1567 nm for both the polarisations. It can be noted that the proposed PI-SSC is stable with the wavelength variation due to the strong coupling between the lower silicon NW and the upper multi-layer. For the proposed polarisation combiner, variations of the coupling length with the wavelength are shown in Fig. 13(b). It can be noted that the coupling length of the quasi-TE mode is varied from 10559 μm to 7209 μm with the wavelength increased from 1500 nm to 1600 nm, while the coupling length of the quasi-TM mode is changed from 384.5 μm to 319.7 μm. The large difference between these two coupling lengths can always enable a polarisation combiner with the wavelength change as large as 100 nm. As the length of the polarisation combiner, *L* = ${L}_{{\rm{c}}}^{x}$, it can also be noted from Fig. 13(b) that the combiner length was reasonable constant in between 336 μm and 356 μm for the wavelength variation of 30 nm from 1566 nm to 1536 nm.

**Figure 14:**
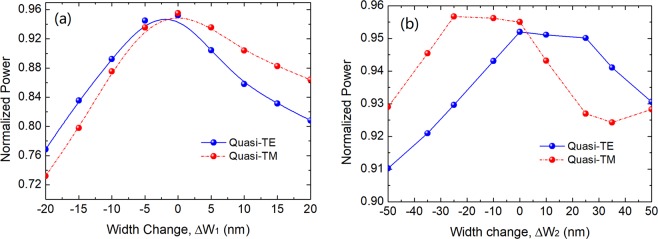
Variations of the normalised power with the width changes of **(a)** lower-nanowire width, W_1_ and **(b)** upper-array width, W_2_ for both the TE and TM polarisations.

The thicknesses of the silicon and silica layers can be accurately controlled by using the plasma enhanced chemical vapour deposition (PECVD) process, but the fabrication error of the lithography process may cause the width errors for both the lower and upper waveguides in the range of ±20 nm for most foundries. Therefore, we calculate variations of the normalised power with the width changes of the lower-NW width, W_1_ and the upper-array width, W_2_ for both the TE and TM polarisations, as shown in Fig. 14. It can be noted from Fig. 14(a) that with the width change of ΔW_1_ = ±20 nm, the deteriorations of the normalised power are less than 0.18 and 0.22 for the quasi-TE and quasi-TM modes, respectively. It can also be noted from Fig. 14(b) that the normalised powers are larger than 0.91 and 0.93 with the width change of ΔW_2_ = ±50 nm for the quasi-TE and quasi-TM modes, respectively. Hence, the proposed PI-SSC is more tolerant to the width change of the upper layered-waveguide due to its wider width of 6 μm. If the two waveguides are misaligned at their centres, the power loss is expected to be very small. This is because, if we move the lower NW below a much wider layered-guide, the resulting change in the supermodes due to that shift is very small. We have also calculated the variations of the normalised power with the misalignment error and results prove that the normalised power is almost constant with the misalignment error of ±50 nm.

### Tapering multi-layer

**Figure 15:**
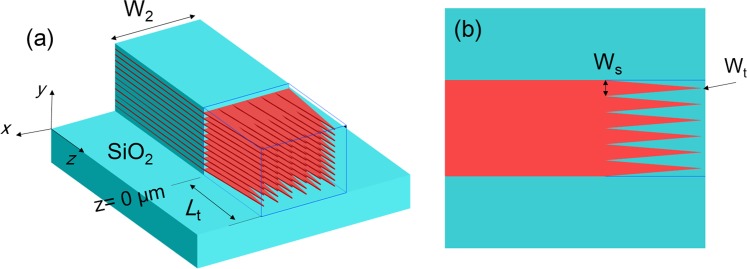
**(a)** Schematic of the multi-layer tapers. **(b)** Top view of the multi-layer tapers.

As stated above, the output waveguide of the proposed PI-SSC is based on the multi-layer structure, which may have some challenges in polishing the end-face. In this case, we propose to horizontally taper the multi-layer in several sections after the quasi-TM mode has coupled to the multi-layer guide, as shown in Fig. 15(a). These tapers are identical with the same length of *L*_t_. The top view of the multi-layer tapers is shown in Fig. 15(b). The width of each taper is linearly narrowed from W_s_ to the taper-end width, W_t_. There are two advantages by introducing these multi-layer tapers: (i) field profile of the output mode will become more smooth Gaussian-like field, which can be expected to couple better to an SMF or a lensed fibre; (b) it will mostly be SiO_2_ at the end face of the SSC, so it would be easy to polish the end face compared with the wider multi-layer guide. The coupling loss can be further reduced by implementing the index-matching oil as the end face is homogeneous.

**Figure 16:**
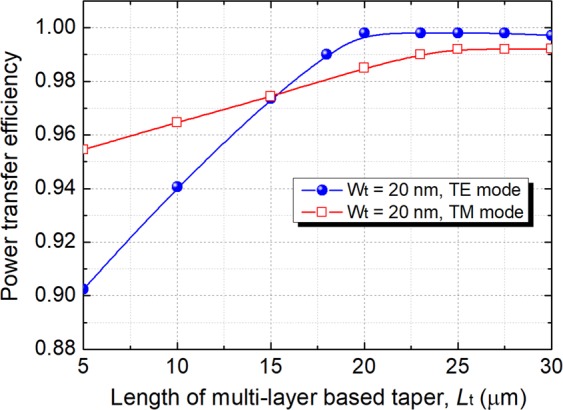
Variations of the power transfer efficiency with the length of the multi-layer tapers.

**Figure 17:**
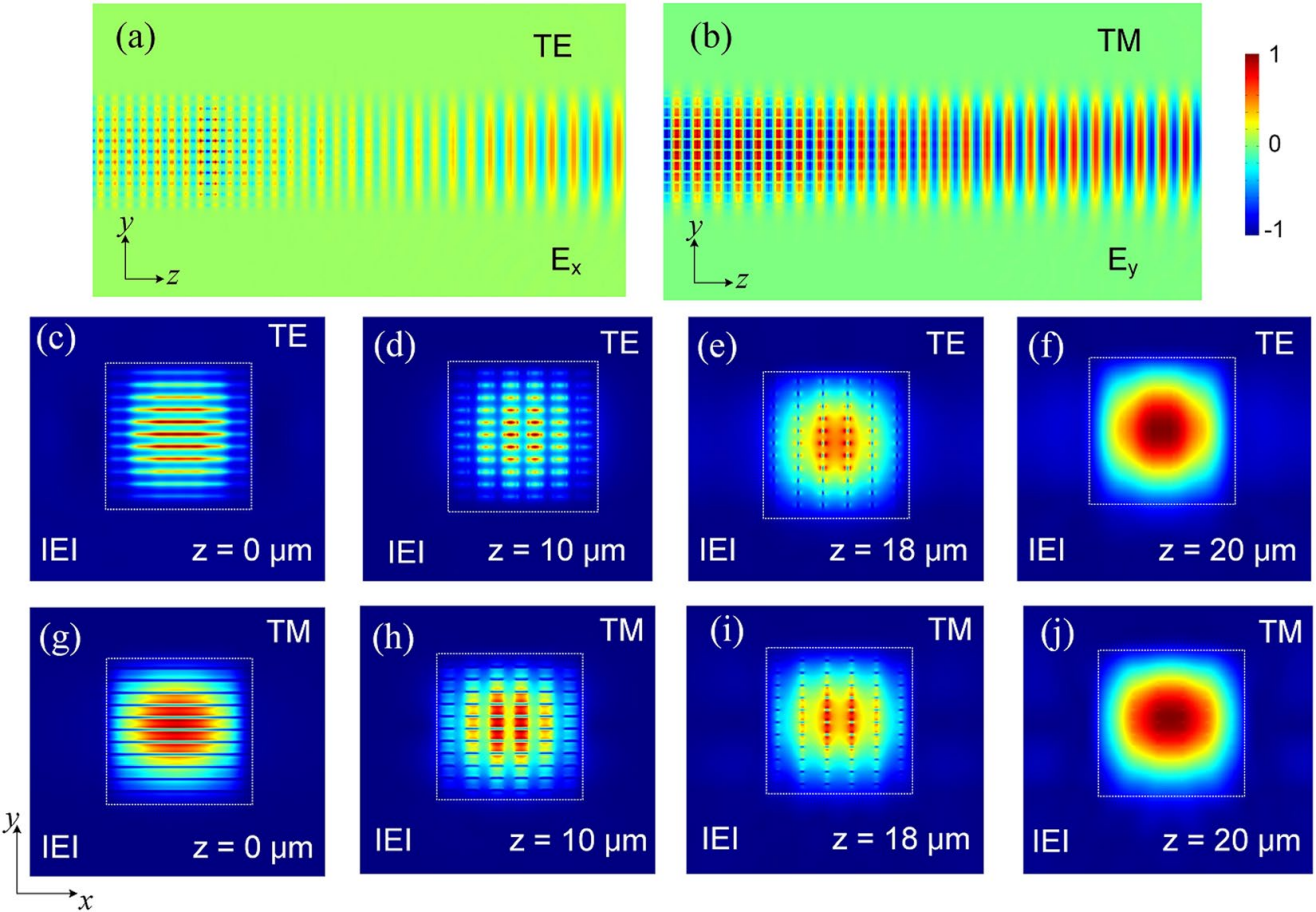
Optical fields of the multi-layer tapers. Propagation fields of the multi-layer taper for **(a)** TE and **(b)** TM modes; **(c**–**j)** are the electric-field intensities along *z* direction for the TE and TM modes, respectively.

Next, the design of the multi-layer tapers is optimised by using the 3D-FV-FDTD method. Variations of the power transfer efficiency with the length of the multi-layer tapers are shown in Fig. 16 for both the quasi-TE and quasi-TM polarisations. In the calculation, the parameters were set as: W_s_ = 1.0 μm and W_t_ = 20 nm. It can be noted from Fig. 16 that the power transfer efficiencies of both the polarisations are increased with the increase of the taper length, *L*_t_. The power transfer efficiency of the quasi-TE mode reaches to 99.8% after *L*_t_ = 20 μm and that of the quasi-TM mode is greater than 98.5%. The optical fields of the optimised multi-layer tapers for *L*_t_ = 20 μm are shown in Fig. 17. Propagation fields of the multi-layer tapers are shown in Fig. 17(a,b) for the quasi-TE and quasi-TM polarisations, respectively. It can be noted that the fundamental modes of both the polarisations are completely converted from the “multi-layered” field to the smooth Gaussian-like field and then coupled to an SMF or a lensed fibre. The electric-field intensities along *z* direction are shown in Fig. 17(c–j) for the quasi-TE and quasi-TM modes, respectively. The mode evolution across the x-y cross-section also proves that the output mode fields transferred from the “multi-layered” Gaussian fields to the smooth Gaussian-like fields for both the polarisations. The coupling efficiencies between the output Gaussian-like fields and the SMF/lensed fibre are also calculated for both the polarisations. When coupling to an SMF, the coupling efficiency at Junction 2 can be enhanced from 56.1% to 62.1% for the quasi-TE polarisation and that for the quasi-TM polarisation is improved from 59.6% to 61.8% by using these multi-layer tapers. The coupling efficiency is also increased by 0.6% and 0.4% for the quasi-TE and quasi-TM polarisations, respectively when coupling to the lensed fibre mentioned above.

## Conclusion

In conclusion, we have proposed and optimised a CMOS-compatible PI-SSC incorporating the phase-matched multi-layer, which consists of an untapered-tapered-untapered silicon NW and two multi-layer sections on an SOI platform. The PI-SSC has been optimally designed by using an efficient algorithm, combining the **H**-field based VFEM and the LSBR method. The vector modal field profiles and confinement factors have been calculated by using the VFEM. The phase-matching conditions for both the isolated and composite waveguides have been studied. Subsequently, the supermode coefficients and power transfer efficiency have been calculated by using the LSBR method. With an eleven-layer based PI-SSC, the coupling losses between a silicon NW and an SMF can be reduced to 2.72 dB and 2.45 dB for the quasi-TE and quasi-TM polarisations, respectively. However, using a lensed fibre of radius 2 μm instead of an SMF, the coupling losses can be significantly reduced to 0.34 dB and 0.25 dB for both the quasi-TE and quasi-TM polarisations, respectively. The 1 dB bandwidth is 41.4 nm for both the polarisations, which could be further improved by using a smaller separation between the lower silicon NW and the upper multi-layer. A smaller separation will also make the design more compact, more resistant to fabrication tolerances, but the power loss would increase slightly. The coupling efficiency was further improved by tapering the multi-layer, which can also simplify polishing the end-face. Although the proposed PI-SSC needs an additional mask and a multi-deposition step, the enhanced in-plane coupling efficiency would allow to overcome one of the key challenge remaining, efficient in and out coupling of a silicon PIC.

## Methods

The vector modes and supermodes of the isolated and composite waveguides are calculated by using a rigorous **H**-field based full-vectorial finite element method (VFEM). The confinement factors of the supermodes are studied and phase-matching conditions are determined by using the VFEM. The supermode coefficients and power transfer efficiency are studied by using the least squares boundary residual (LSBR) method. The coupling losses between the proposed polarisation-independent spot-size converter (PI-SSC) and fibres are calculated by using the LSBR.

The LSBR method has been proved to be a powerful approach to study power transfer in nonidentical and strongly coupled waveguide structures. For a strong discontinuity interface, such as the end of the PI-SSC and a fibre interface, the LSBR method can be used to estimate both the transmission and reflection coefficients by imposing the continuity of the transverse electric and magnetic fields at the junction interface in a least-squares sense. The LSBR method looks for a stationary solution to satisfy the continuity conditions in a least squares sense by minimizing the error energy functional, *J*, as given by^27^

2 $$J=\int {|{E}_{t}^{I}-{E}_{t}^{II}|}^{2}+\alpha \cdot {Z}_{0}^{2}{|{H}_{t}^{I}-{H}_{t}^{II}|}^{2}d{\rm{\Omega }}$$

where ${E}_{t}^{I}$, ${H}_{t}^{I}$ and ${E}_{t}^{II}$, ${H}_{t}^{II}$ are the transverse electric and magnetic fields in sections I and II, respectively. *Z*_0_ is the free-space impedance, Ω is the junction interface, and *α* is the dimensionless weighting factor to balance the electric and magnetic components of the error functional *J*. The LSBR method is more rigorous than the overlap method. Overlap method is a simpler method, which considers orthogonality of the modes and can find coefficient of each transmitted modes. It cannot consider radiated modes, as they are no longer orthogonal with the guided modes and also does not consider the reflected modes.

For the mode calculations of a waveguide with 2-D confinement, we do not need to solve a 3D vector Helmholtz equation but a 2-D wave equation, which we have carried out and the results are exact to the limit. But, for the proposed structure including the junctions, taper, S-bend section, we have used 3D wave equation via the 3D full-vectorial finite difference time domain (3D-FV-FDTD) method. It may be possible to solve the entire structure with the bending regions as a single problem by using the 3D-FV-FDTD method. But, a high-performance workstation is needed and it would be too expensive computationally. In this case, the taper length of section II is optimised by using the commercial COMSOL Multiphysics. The characteristics of the S-bend layered-waveguide and tapering multi-layer are studied by using the 3D-FV-FDTD method.
